# Australia’s overseas development aid commitment to health through the sustainable development goals: a multi-stakeholder perspective

**DOI:** 10.1186/s12992-019-0507-5

**Published:** 2019-11-21

**Authors:** Claire E. Brolan, Christopher A. McEwan, Peter S. Hill

**Affiliations:** 1 Centre for Policy Futures, The University of Queensland, Brisbane, Australia; 2 School of Public Health, The University of Queensland, Brisbane, Australia

**Keywords:** Sustainable development goals, SDGs, SDG 3, Health and development, Australia, Overseas development aid, ODA, Indo-Pacific, Health policy, Governance

## Abstract

**Background:**

In 2018, the Australian Government, through a Senate-led Parliamentary Inquiry, sought the views of diverse stakeholders on Sustainable Development Goal (SDG) implementation both domestically and as part of Australia’s Overseas Development Assistance (ODA) program. One hundred and sixty-four written submissions were received. The submissions offered perspective and guidance from a rich cross-section of those involved, and with keen interest in, Australia’s ODA-SDG commitment. This article identifies and explores the submissions to that Inquiry which placed impetus on Australia’s ODA-SDG and health and development nexus. It then compares how the synthesized views, concerns and priorities of selected Inquiry stakeholders align with and reflect the Australian Government’s treatment of SDG 3 in its SDG Voluntary National Review (VNR), as well as with the final Inquiry report summarizing submission content.

**Results:**

Four key themes were synthesized and drawn from the thirty-one stakeholder submissions included in our analysis. Disconnect was then found to exist between the selected stakeholder views and the Australian Government’s SDG-VNR’s treatment of SDG 3, as well as with the content of the Parliamentary Inquiry’s final report with respect to the ODA-SDG and health and development nexus.

**Conclusions:**

We situate the findings of our analysis within the wider strategic context of the Australian Government’s policy commitment to “step up” in the Pacific region. This research provides an insight into both multi-stakeholder and Federal Government views on ODA in the Indo-Pacific region, especially at a time when Australia’s Pacific engagement has come to the forefront of both foreign and security policy. We conclude that the SDG agenda, including the SDG health and development agenda, could offer a unique vehicle for enabling a paradigm shift in the Australian Government’s development approach toward the Pacific region and its diverse peoples. This potential is strongly reflected in stakeholder perspectives included in our analysis. However, study findings remind that the political determinants of health, and overlapping political determinants of SDG achievement, will be instrumental in the coming decade, and that stakeholders from different sectors need to be genuinely engaged in SDG-ODA policy-related decision-making and planning by governments in both developed and developing countries alike.

## Introduction

**Fig. 1:**
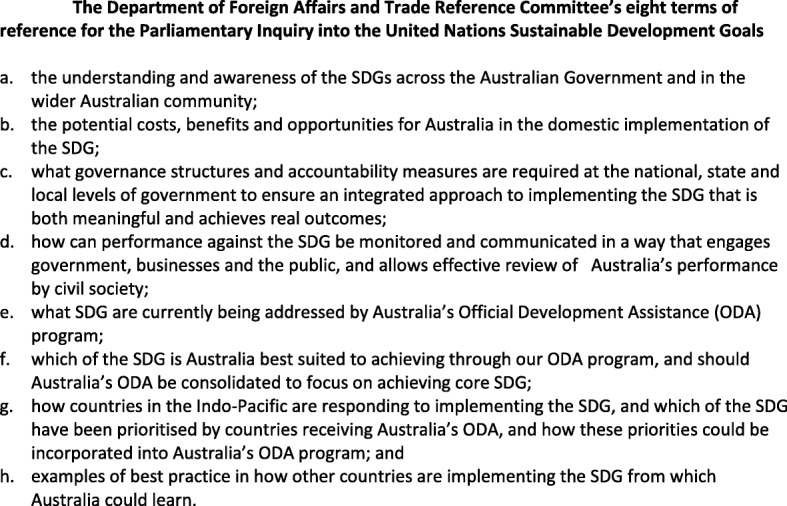
The Department of Foreign Affairs and Trade Reference Committee’s eight terms of reference for the Parliamentary Inquiry into the United Nations Sustainable Development Goals

In 2018, the Australian Government conducted a Parliamentary Inquiry into the implementation of the United Nations (UN) Sustainable Development Goals (SDGs) in Australia and as part of Australia’s Overseas Development Aid (ODA) progra m[1]. The inquiry sought the views of government and non-government actors and institutes, and the general public, with 164 submissions received from a range of actors and sectors. The Parliamentary Inquiry sought responses to eight terms of reference, including scoping questions on how the SDGs might shape Australia’s ODA commitments (Fig. 1) [2]. This article focuses on support for the SDGs within Australia’s development role, exploring the rich experience, knowledge and voice on Australia’s health, development and ODA-SDG nexus within those submissions. It then examines whether those multi-sectoral insights and recommendations are consistent with the Australian Government’s treatment of SDG 3 (Ensure healthy lives and promote wellbeing for all at all ages) in the ODA context in its first Voluntary National Review (VNR) on SDG Implementation of June 2018, as well as with the Foreign Affairs and Trade References Committee’s (FATRC) summary of the Inquiry submissions released in 2019 [3, 4]..

The research – and findings – identify and integrate a number of key policy topics of contemporary import and relevance to the Australian Government at a time of post-election transition. The findings and analysis connect Australia’s SDG health and development commitments in the Indo-Pacific region with Australian ODA policy, and situate this nexus in the wider context of Australia’s new strategy of “stepping-up” in the Pacific [5]..

## Background

Australia was one of 193 countries at the high-level UN Sustainable Development Summit in New York in September 2015 that unanimously committed to achieve the 2030 Agenda for Sustainable Development and its 17 Sustainable Development Goals (SDGs), including the health goal SDG 3 (Ensure healthy lives and promote wellbeing for all at all ages )[6]. Thus, SDG implementation is the shared responsibility of all countries at all stages of development, including OECD nations like Australia, and SDG 17 (Strengthen the means of implementation and revitalize the global partnership for sustainable development) is explicit in the multifaceted role developed countries must play in supporting developing countries, and regions, fully realize their SDG potentia l[7]. While the SDG agenda aims to leverage global commitment on a set of targets to reach by 2030, it lacks a shared strategy of implementation; the choice of planning and action is left to UN Member State s[8]. Well-resourced countries are grappling with the machinations of integrating the SDGs into national economic, social and environmental policy, and, of particular interest to this paper, into their international development program s[9–11]. Indeed, political commitment (that includes financing) and integrated, systems approaches to SDG policy, planning and implementation will be key[12, 13]. The Australian context is no different [14]..

The Australian Government initially signaled its support for the SDGs in the context of its ODA program, when it released its 2017 Foreign Policy White Paper: “In working with partners to achieve the SDGs, Australia will use its [ODA], including through aid for trade, to catalyze sustained and inclusive economic growth to help reduce poverty”. ([15] (p88)) In June 2018, Australia’s first VNR on SDG implementation was released, affirming the Australian Government’s September 2015 pledge to achieve the SDGs domestically, as well as support Indo-Pacific countries’ SDG achievement through Australia’s international development and humanitarian assistance program [3]. Following the government’s release of Australia’s SDG-VNR, in late 2018 Prime Minister Scott Morrison announced Australia would “step up its Pacific engagement” to forge a “new chapter in relations with our Pacific family”, including establishing five new diplomatic missions in the Pacific and the creation of the Office of the Pacific within the Department of Foreign Affairs and Trade (DFAT) [5]..

Australian Government onus on “stepping-up in the Pacific” continues with the re-election of Prime Minister Morrison at the May 2019 Federal election, and remains focused on enhancing strong Australian-Pacific economic and security partnerships, and stronger social, educational and cultural “people-to-people” links [16]. However, the Australian Government’s interest and impetus in the Pacific, and in the broader Indo-Pacific region, is a point of ongoing scrutiny. In particular, what constitutes the Indo-Pacific region (and whether it is a helpful geographical framing), and the enduring securitization paradigm that appears to be driving and informing Australia’s aid efforts in the Indo-Pacific, and specifically within the Pacific region [17–20]..

China’s increased development engagement with Pacific Island governments, although it does not surpass Australia’s current development investment, has undoubtedly contributed to growing unease within the Australian Government as to Chinese influence (and the proactivity of this influence) in a traditionally Australian-dominated geopolitical sphere [20, 21]. Hameiri argues, “This is mainly because of the perception that China’s engagement with the region is part of a broader intensification of geopolitical competition with the USA” [22]. Hence, foregrounding Australia’s development role in and with its “Pacific family” is making news headlines,[23] and the government’s concurrent investment in health security in the Pacific is interconnected [24, 25]. The latter development policy commits the Federal Government to strengthen public health systems in Southeast Asia and the Pacific, with a view to “promote economic growth and development, protect Australia and Australians against the impact of these health threats, and decrease the risk of economic shocks arising from the suspension of trade and movement of people” [26]..

Yet, the public admonishment of the Australian Government’s development motives and self-interest in the Pacific region, by Pacific leaders, at the Pacific Islands Forum meeting in Tuvalu in August 2019, highlights Pacific self-determination and growing regional solidarity [23, 27–29]. It also highlights the real frustration with the Australian Government’s stance, both at home and abroad, on climate change issues – while for Australia’s Pacific Island “family”, the consequences of climate change and rising sea-levels are a more literal threa t[30, 31]. Recent exchanges in the Pacific Forum meetings have raised criticism of the Australian Government’ sensitivity to mutually respectful, bilateral Pacific relationship s[23, 27, 32]. The recalibration of Australian priorities will benefit Pacific Island countries and territories, and likewise more effectively serve Australia’s regional soft power ambitions.

This article examines the content of the written submissions to the 2018 Australian Parliamentary Inquiry into the UN SDGs seeking to explicate and explore the responses pertinent to health within Australia’s ODA-SDG context. The article synthesizes the key ODA-SDG and health priorities raised by the Inquiry’s respondents and juxtaposes these stakeholder perspectives with the content of the Australian Government’s first SDG-VNR, as well as the FATRC report of its findings on the Parliamentary Inquiry.

## Methods

The objective of this research is twofold. We first sought to explore the views of diverse stakeholders on Australia’s ODA-SDG implementation nexus, with a particular interest in Australia’s approach to health and development in the Indo-Pacific region. This investigation uses the publicly available written submissions to the Australian Parliamentary Inquiry into the UN SDGs of 2018 as the data set for documentary analysi s[33]. That Inquiry, led by the FATRC in the Senate Department (following a Senate referral on 4 December 2017), comprised an online public submission process, and public meetings in Sydney, Melbourne and Canberra. Our second research objective is to compare the findings that emerge from our thematic analysis of the selected written submissions with the Australian Government’s treatment of SDG 3 in the ODA context in its first SDG-VNR of June 2018, as well as with the FATRC summary of the Inquiry submission content released in 201 9[3, 4]. We note that this study’s analysis was undertaken prior to the FATRC releasing its report into the Senate Inquiry into the UN SDGs in February 2019, and thus our analysis is not influenced by the content or findings of that report.

To identify which written submissions to the Australian Parliamentary Inquiry into the UN SDGs of 2018 provided the most detail on the crosscutting topics of ODA, health and development to include in this analysis, and guided by the methodology in a previous documentary review of parliamentary inquiry responses, [34] we reviewed all 164 written submissions to the UN SDG Inquiry. These were accessed from a publicly available Australian Parliamentary website, where written submissions were posted after formal acceptance by the FATRC delegated to lead the UN SDG Inquiry [33, 35]. The 164 submissions totaled 1983 pages, with over two-thirds (69%) prepared by non-government actors (Table 1). The average length of each submission is 12 pages: the shortest is one page in length (made by two individual Inquiry respondents and one non-government actor), while the longest is by the international tobacco company Philip Morris International Australia, New Zealand and Pacific (PMI) at 169 pages. A 90-page submission from the Sustainable Development Solutions Network (SDSN) Australia-New Zealand-Pacific was the next largest.

**Table 1:** A snapshot of the parties that made submissions to the Australian Parliamentary Inquiry into the UN SDGs

Inquiry respondents	Study %
Non-government actors	69% (*n* = 114)
i.e. submissions made by civil society organizations, community networks, peak bodies, educational entities (schools or universities), business and industry, or the private sector more broadly	
Government organisations or agencies	12% (*n* = 19)
i.e. submissions made by Federal, state/territory and local government or government delegations	
Individuals	18% (*n* = 30)
i.e. submissions made by members of the public	
Joint	> 1% (*n* = 1)
i.e. submissions made by government and non-government actors	

Each of the 164 submissions were read by CEB, to assess whether the submission content focused on both the Australian Government’s ODA program *and* health and wellbeing (Table 2). The content of 31 submissions were identified as relevant to this study and included for thematic analysis, as described by Attride-Stirling (Table 2 )[36]. Several further readings of these 31 papers led to identification of emergent themes building on eight themes corresponding to the Inquiry’s Terms of Reference. Emergent thematic findings were iteratively discussed between CEB and PSH, and final thematic findings were confirmed by both team members to ensure inter-rata reliability and study rigor. CM assisted in revising several manuscript drafts.

**Table 2:** Submissions in this study that include focus on the Australian Government’s Overseas Development Aid program, health and wellbeing, and the Sustainable Development Goals^1^

No.	Stakeholder Name	Submission No.	Description	Role, Aim or Vision
1	Burnet Institute	#10	Burnet Institute is unique in Australia in being both a medical research institute accredited by the National Health and Medical Research Council and a development non-government organization (NGO) fully accredited by DFAT.	The Institute’s vision is to achieve equity through better health, and its mission for next five years is to achieve better health for vulnerable communities in Australia and internationally by accelerating the translation of research, discovery and evidence into sustainable health solutions.
2	PhD Candidate, Associate Professor and Professor (3 Individuals)	#17	Three sustainable development and SDG specialist researchers from the University of New South Wales (UNSW).	The authors of this submission support the Australian Government’s integration of the SDGs into its ODA program
3	Oxfam Australia	#18	Oxfam Australia is an independent, not-for-­profit, secular development agency and a long-term development partner of the Australian Government.	Oxfam Australia’s vision is of a just world without poverty and it undertakes long-term development projects, provides emergency response during disaster and conflict, undertakes research and advocacy to advance the right of poor and marginalised people, and promotes fair trade.
4	Vision 2020 Australia	#19	Vision 2020 Australia is part of VISION 2020: The Right to Sight, a global initiative of the World Health Organization and the International Agency for the Prevention of Blindness. Vision 2020 Australia is the national peak body for the eye health and vision care sector, and represents 50 member organisations involved in local and global eye health and vision care, health promotion, low vision support, vision rehabilitation, eye research, professional assistance and community support.	Vision 2020 Australia’s aims to ensure that eye health and vision care remains high on the health, ageing, disability and international development agendas of Australian governments.
5	World Vision Australia	#25	World Vision Australia is part of World Vision, a worldwide community development organisation that provides short-term and long-term assistance to 100 million people worldwide (including 77 million children). World Vision Australia partners with the Australian Government to deliver the Australian ODA program and engages in dialogue with the Australian Government on matters of policy and practice	The vision of World Vision is ‘for every child, life in all its fullness; our prayer for every heart, the will to make it so.’
6	Family Planning NSW	#32	Family Planning NSW is the leading reproductive and sexual health (RSH) agency in Australia with over 90-year’s history. We are DFAT accredited and provide international development activities in RSH across the Indo-Pacific region.	Family Planning NSW provides RSH services, professional education and training, and research and evaluation in Australia, focusing in NSW.
7	Doctors for the Environment Australia	#33	Doctors for the Environment Australia is a voluntary organisation of medical doctors in all Australian states and territories, and effectively functions as a public health organization.	Doctors for the Environment Australia works to address local, national and global health effects caused by damage to the earth’s environment in line with the medical profession’s proud community service record.
8	The Fred Hollows Foundation	#36	The Fred Hollows Foundation is a leading international NGO working to restore sight, eliminate avoidable blindness and ensure Indigenous Australians exercise their right to good health. We work with local partners and governments in Australia and in more than 25 countries around the world to prevent and treat the major eye diseases causing avoidable blindness: cataract, trachoma and diabetic retinopathy.	The Fred Hollows Foundations works to meet the challenge that without better funding and access to eye care services, the number of people who are blind will triple from 36 million to 115 million by 2050, and these 36 million people, 4 out of 5 suffer from conditions that can be treated or prevented
9	Global Partnership for Education (GPE) Secretariat	#41	The GPE is a global multi-stakeholder partnership and fund for education and is the only fund which focuses solely on ensuring equitable, quality education for all through an inclusive systems-strengthening and results-based approach.	GPE mobilizes global funding, knowledge and political will; and acts locally, locking together better planning, more financing and mutual accountability to build strong education systems and better schools.
10	University of Sydney	#52	Higher education and tertiary research institute in New South Wales (NSW), Australia.	To benefit the people of NSW, Australia and the wider world.
11	Sustainable Development Solutions Network (SDSN), Australia/ Pacific	#55	SDSN Australia/Pacific is part of the SDSN, is a global network of universities and knowledge institutions that mobilises scientific and technical expertise to promote practical problem solving for sustainable development, including the implementation of the SDGs. SDSN has been operating since 2012 under the auspices of the UN Secretary-General, and has over 700 member institutions around the world.	The Australia/Pacific regional network of SDSN was established in 2013 and is hosted by Monash University. It works with the 30 SDSN members in Australia, New Zealand and the Pacific, as well as with many other partners from across all sectors, around a number of SDG-related areas.
12	Department of Foreign Affairs and Trade (DFAT), Australian Government	#60	DFAT is the department of the Government of Australia responsible for foreign policy, foreign relations, foreign aid, consular services, and trade and investment.	DFAT supports and operationalises the Australian Government’s integration of the SDGs into its ODA program
13	The International Sexual and Reproductive Health and Rights Consortium	#62	The International Sexual and Reproductive Health and Rights Consortium (The Consortium) is an Australian NGO partnership.	The Consortium champions universal access to sexual and reproductive health and rights, and NGO consortium partners believe that all people should have the freedom to make their own decisions about their bodies and their lives.
14	Cardno International Development	#64	Cardno International Development is a long-term DFAT partner, and works with DFAT to design and implement government-funded programs	The role of Cardno International Development is to deliver over 50 DFAT programs in 20 countries in the Asia-Pacific region.
15	Victoria University’s Timor-Leste Reference Group	#65	Higher education and tertiary research institute in Victoria, Australia, with unique and enduring relationship with Timor-Lest.	In 2017, Victoria University released its Green Paper that led to the development and implementation of a *Victoria University Timor-Leste Strategic Plan (2018–2020)* demonstrating unequivocal institutional commitment to clear, considered and measurable engagement with Timor-Leste with a continuing and renewed focus on sustainable development through an SDG lens
16	Economics & International Relations student (Individual)	#69	Young Australian student from the University of Adelaide with strong interest in international affairs.	Individual student keen to see Australia harness the opportunities presented by the SDGs to make the world a better place by 2030 and beyond.
17	Results International Australia	#71	Results International Australia is part of an international, non-partisan and non-profit organisation that has been working in Australia for 30 years through a combination of staff-led and grassroots-driven advocacy, with service delivery in 37 countries.	Results International Australia works with federal parliamentarians and through the media to generate public and political will to end poverty, and focuses its advocacy on global health issues such as tuberculosis (TB), HIV, malaria, polio, child health, vaccines and nutrition, as well as education and microfinance.
18	Marie Stopes International Australia	#82	Marie Stopes International Australia is a leading global provider of SRH services and key advocate for reproductive rights	Marie Stopes International Australia transforms the lives of women and girls by providing them with reproductive choice, which includes access to SRH education, counselling, modern forms of contraception, STI and HIV testing, cervical cancer screening and safe abortion.
19	Save the Children Australia	#84	Save the Children Australia is part of Save the Children, a leading independent international organisation for children, and works toward the agency’s vision for child rights in Australia and more than 120 countries across the globe.	Save the Children Australia’s vision is a world in which every child attains the right to survival, protection, development and participation.
20	Commonwealth Scientific and Industrial Research Organisation (CSIRO)	#85	CSIRO is an independent Australian federal government agency responsible for scientific research.	CSIRO’s chief role is to improve the economic and social performance of industry for the benefit of the community by solving the greatest challenges through innovative science and technology
21	UNICEF Australia	#87	UNICEF Australia is a national committee of UNICEF, a multilateral organisation that works in over 190 countries to promote and protect the rights of children.	UNICEF Australia advocates for the rights of all children and works to improve public and government support for child rights and international development.
22	International Women’s Development Association (IWDA)	#98	IWDA is the leading Australian agency entirely focussed on women’s rights and gender equality in the Asia Pacific region, and is international, feminist and independent.	IWDA’s vision is gender equality for all and its purpose is to advance and protect the rights of diverse women and girls.
23	Public Health Association of Australia (PHAA)	#99	PHAA is the principal NGO for public health in Australia	PHAA works to promote the health and well-being of all Australians by to driving better health outcomes through increased knowledge, better access and equity, evidence informed policy and effective population-based practice in public health, and its efforts are advanced by its vision for a healthy Australia: health is a human right, a vital resource for everyday life, and key factor in sustainability.
24	Australian Parliamentary Group on Population and Development (APGPD)	#116	The APGPD are part of a global network of parliamentary groups on population and development that similarly connect parliamentarians across party lines and country borders to work cooperatively on their shared vision.	The APGPD works across party lines to champion women’s empowerment, break down gender discrimination, and advocate for women’s right to access quality reproductive health services through Australia’s foreign policy engagement, and since 2000 APGPD’s vision and remit has been guided by the Millennium Development Goals, and since 2015, the SDGs, as outlined in the Group’s ‘Rules and Objectives’.
25	Monash University	#120	Higher education and tertiary research institute in Victoria, Australia.	To benefit the people of Victoria, Australia and the wider world.
26	Global Citizen Australia	#122	Global Citizen is an Australian-grown international advocacy organisation dedicated to ending extreme poverty by 2030.	Global Citizen is building the world’s largest movement for social action towards ending extreme poverty through organising massive global campaigns to amplify the actions of Global Citizens from around the world and ensure that poverty reduction is on the agenda for the world’s key political moments.
27	Australian Council for International Development (ACFID)	#135	ACFID is the peak body for Australian NGOs involved in international development and humanitarian action.	ACFID’s vision is of a world where all people are free from extreme poverty, injustice and inequality and where the earth’s finite resources are managed sustainably, and ACFID’s purpose is to lead and unite its members in action for a just, equitable and sustainable world.
28	Australian Human Rights Commission	#138	The Commission is Australia’s National Human Rights Institution (NHRI) and is accredited as an ‘A status’ NHRI under the United Nation’s Paris Principles.	The Commission has a statutory power to promote and protect human rights under the *Australian Human Rights Commission Act 1986*, and as an independent statutory agency, the Commission has functions including education, awareness raising and inquiring into and conciliating complaints of unlawful discrimination and breaches of human rights.
29	Department of Health, Australian Government	#143	The Department of Health has a diverse set of responsibilities, including supporting Australia’s world class health system, supporting universal and affordable access to high equality medical, pharmaceutical and hospital services, while helping people to stay healthy through health promotion and disease prevention activities.	The Department of Health is committed to the implementation of the 2030 Agenda for Sustainable Development and its corresponding 17 SDGs. The Department’s Vision statement is ***Better health and wellbeing for all Australians, now and for future generations*, and it seeks to achieve** its Vision through strengthening evidence-based policy advice, improving program management, research, regulation and partnerships with other agencies, consumers and stakeholders.
30	Australian Centre for International Agricultural Research (ACIAR)	#157	ACIAR is Australia’s specialist international agricultural research for development agency, and is an independent statutory agency within the foreign affairs portfolio.	ACIAR’s mission is to achieve more productive and sustainable agricultural systems, for the benefit of developing countries and Australia, through international agricultural research partnerships.

^1^The content in Table 1 was directly obtained from each stakeholder’s submission and where this detail was not included in the submission, the stakeholder’s website was accessed for elucidation

Following Weitzman’s [37] guidance on the use of computer software in qualitative research, a number of possible analytical strategies were initially identified among research team members for interrogation of the qualitative data set, and their use and benefits discussed. Analysis software (such as NVivo) were not used because it was agreed that quantitative content analysis and data visualization would not necessarily add analytical value, and had the potential to detract or distort the contextual richness and meaning of the stakeholder views and voices in the individual submissions. There was also concern that coding through a software package might distance the researchers from this data set [38]. Manual coding and analysis of the submissions was thus undertaken by CEB and a selection of 5% of submissions checked by PSH, with text-based management of the data (and thematic subsets of data) organized in tabular form using Microsoft Excel and Microsoft Word.

In terms of the second research objective, once the thematic findings from the documentary analysis were finalized, the research team turned their attention to comparing those findings on the priorities and issue areas identified by the selected Inquiry stakeholders on the Australian ODA-SDGs-health and development nexus with the Australian Government’s treatment of this same nexus within Australia’s SDG-VNR of June 2018. Research team members separately reviewed the 130-page VNR document, especially focusing on that document’s treatment of health in the ODA context in the chapter on SDG 3 (Good health and wellbeing), and compared and contrasted the Australian Government’s position and priorities in the VNR with this study’s preliminary findings. The provisional findings of this comparison were presented to a workshop of policy analysts and academics, which allowed the research team members to discuss and finalize key comparative findings. A similar process was engaged to compare the content of our analysis of respondents’ ODA-SDG-health and development priorities with the summary offered by FATRC’s final report on the UN SDG Inquiry.

## Results

### Part 1 - findings of the documentary analysis

Thirty-one of the 164 written submissions to the Parliamentary Inquiry into the UN SDGs of 2018 were identified to have a strong narrative and contextual focus on both the Australian Government’s ODA program *and* health and wellbeing, and were thus subject to thematic analysis. These 31 written submissions totaled 583 pages. When the content of these submissions was subject to thematic analysis, four broad themes on ODA, the SDGs and health and wellbeing emerged:

1. Different characterizations of health in the SDG context by Inquiry stakeholders;
2. Importance of governance and multi-sectoral planning for integrating SDG implementation into Australia’s ODA program;
3. Identification of health priorities for Australia’s ODA program, especially in the Indo-Pacific region; and
4. Financing for regional and global health (and the SDGs broadly).

### Theme 1: different characterizations of health

We found the respondents characterized health in three distinct ways. First, Australia’s pursuit of good health and wellbeing in the Indo-Pacific context is seen as critical for broader regional development beyond health. Second, there is a sense among some respondents that greater onus on sexual and reproductive health (SRH) will enable the achievement of the broader SDG framework, again beyond narrower health (SDG 3) attainment. Agencies with a SRH mandate were particularly strong in their advocacy for this position. Finally, two respondents suggest the SDGs present an opportunity for the integration of Australia’s current health and development approach into the more crosscutting, multi-sectoral Planetary Health approach, which these respondents contend has stronger SDG resonance.

#### Advancement of SDG 3 is critical to enable broader indo-Pacific regional development under the SDG agenda

Although SDG 3 achievement should be prioritized for “efficient and effective” SDG implementation [Fred Hollows Foundation], health and wellbeing are the product and outcome of all 17 SDGs. Thus, the pursuit of health and wellbeing in the Indo-Pacific context will require more than siloed focus on SDG 3 achievement:

“A lesson learned from the MDG era is that each global challenge and therefore each of the thematic [SDGs] are necessarily interlinked, and success for one goal cannot be complete without investment in others. Health, gender and education… are important cross-cutting issues…” [Global Partnership for Education].

The Department of Health (Australian Government) also emphasizes health’s intersection across the SDG framework: “Good health is both a pre-condition and an outcome of the 2030 Agenda, as the SDGs are closely aligned to the social, economic and environmental determinants of health”.

#### Sexual and reproductive health (SRH) underpins the success of all 17 SDGs

In terms of particular intersections, several respondents specify that “Gender equality is an enabler and accelerator for all the SDGs” [International Women’s Development Association] and “The right to family planning enables fulfilment of other human rights, such as gender equality, by allowing women to access education and employment, increase their negotiating power and raise their socioeconomic status” [Marie Stopes International Australia]. Alternatively, the International Sexual and Reproductive Health and Rights Consortium distinguishes that gender equality and the achievement of sexual and reproductive health *and rights* is crucial to broader SDG achievement:

“A key driver of gender equality is the realization of sexual and reproductive health rights by women and girls. For women to be equal, healthy, educated, financially secure, and live free from violence, they need to be able to make informed decisions about their own bodies.”

#### Coalescence with planetary health

Following the integrated nature of health (including SRH) as a pre-condition and outcome of the entire SDG agenda, Planetary Health - “the health of human civilization and the nature systems on which they depend” - is proposed by Doctors for the Environment Australia as the necessary lens for Australia’s SDG implementation moving forward. The University of Sydney contends that adopting a Planetary Health approach toward Australia’s aid program could usher in the required transformational approach to SDG implementation in both Australia and the region, as a Planetary Health approach will, crucially, focus on “key systemic drivers that cut across the goals”.

### Theme 2: planning and governance for integrating SDG implementation into Australia’s ODA program

Inquiry respondents included in this study discussed at length the need for strong Australian Government leadership and good governance, policy, and planning processes and practices for integrating the SDGs into Australia’s ODA program moving ahead. Diverse stakeholders called for the Australian Government to develop a SDG implementation plan with coherent ODA strategies and policies, SDG awareness raising, the shift from a siloed to integrated approach to SDG planning by government, and the need for investment in robust data in the Indo-Pacific for a strong evidence base for health and development planning, and SDG roll-out broadly.

#### Australia should be an SDG leader in the indo-Pacific and needs a SDG implementation plan

“Australia has a role to play as a leader and norm setter in the region” argues the Fred Hollows Foundation, especially as “[w]e would expect an affluent country to set an example and adopt a leadership position to progress on the SDGs” [Doctors for the Environment Australia]. The Australian Government is encouraged to strategically leverage the “significant” SDG opportunity “to be a leader on sustainable development in the Indo-Pacific” [World Vision Australia]. For Global Citizen Australia, Australia’s SDG leadership in the region is, surely: “Not only the right thing to do, but it is in Australia’s national interest. Investing in our region will… ensure Australia continues to prosper and be a good neighbour”.

To become a regional SDG leader, a number of stakeholders recommend the Australian Government should explicitly align the country’s ODA policy and planning program with the SDGs for policy coherence: “A siloed, two-track approach to the SDGs should be avoided by ensuring coherence between domestic and international action… The SDGs must be affirmed in the purpose of Australia’s aid program, and integrated across its policies, programs, reporting and performance benchmarks” [World Vision Australia]. Like many others, Global Citizen Australia recommends the government develop a SDG implementation plan “outlining how Australia will achieve the goals, both nationally and through our international development and humanitarian assistance efforts”. For Vision 2020 Australia, the lack of a concrete Sustainable Development strategy that encapsulates the SDGs “would be disastrous for the Australian Government’s ambition to grow into a regional leader”. Oxfam Australia furthers that a SDG implementation plan is also “important for solidifying a long-term, bipartisan and strategic approach to the aid program and for meeting the SDGs, whether individual or multiple goals, targets or indicators, according to the project and need”.

#### Raising public awareness on the SDGs and their significance for Australia and the region

A number of stakeholders consider it problematic that Australia’s international development sector appears to have the greatest SDG awareness. Thus, there is strong advocacy among stakeholders for greater SDG awareness raising, particularly led by the Australian Government who, according to Vision 2020 Australia, “must elevate knowledge of the goals beyond the international development and NGO sector by mainstreaming the SDGs to priorities across government, civil and private sectors in simple and understandable terms”.

For Marie Stopes International Australia, raising domestic awareness of the SDGs should include celebrating and promoting ODA achievements with the Australian public, including ODA health achievements:

“Raising awareness of the SDGs allows the government to highlight the successes already achieved domestically and through the Australian Government’s [ODA] program. For example, in Timor-Leste, where the Australian Government has been the largest partner since 2002, it is estimated that the maternal mortality rate reduced by 75% since 1990. The total fertility rate for women in Timor-Leste has also dropped form 7.8 children per woman in 2003 to 4.2 in 2016, demonstrating that Australia’s contribution to women’s health, empowerment and equality has made a significant difference”.

#### Shifting from siloed health and development planning to an integrated approach

As put by the Commonwealth Scientific and Industrial Research Organization (CSIRO), “Implementing the most transformative actions on the SDGs is complex and will require inter-disciplinary and cross-sectoral effort, but yet there is no ‘best practice’ guide on how to achieve this”. Therefore, “Governance mechanisms for the SDGs should be carefully designed to enable greater policy coherence across different departments and levels of government” [Australian Council for International Development]. Accordingly, we found most stakeholders push the Australian Government to adopt an integrated, multi-sectoral, multi-stakeholder and interdisciplinary approach to SDG planning, including an interdisciplinary approach to health and development challenges:

“Given the nature of the 17 goals and their specific focus areas, there is a risk of adopting a siloed approach to implementation, delegating responsibility for each SDG within discrete policy portfolios. Focusing on specific goals in isolation may have the unintended consequence off reversing or undermining progress in other areas”. [Vision 2020 Australia].

The CSIRO affirms that as the SDGS are interlinked and interdependent, the Australian Government should take a systems view of SDG delivery because “a failure to appreciate the cross linkages will likely lead to conflicts, inefficiencies, waste and cost blow-outs”. The CSIRO goes onto state “a major gap, both technically and also from a governance perspective” exists regarding “[h]ow to assess and manage these cumulative impacts, within and between industry sectors”:

“Australia currently does well in managing single industry sectors and is world leading in many… However, more work needs to be done on integrating between sectors and domains, and there are few examples of effective multi-sector integration and management. There will likely be benefits in developing governance structures that allow meaningful consideration but in a cost-effective manner that does not present additional layers of bureaucracy. Understanding the cumulative impacts between and across industry sectors remains a key Australian and global challenge”. [CSIRO].

For the University of Sydney, “[t]here is a need for a [new] framework to better understand and address the complex interlinkages between the social, environmental and governance challenges facing us”. The University of Sydney continues that the SDGs thus offer the Australian Government “a unique opportunity to take an interdisciplinary, cross-sector approach to solving big complex challenges” because the SDGs “are inclusive and diverse enough to be able to speak the language of the majority, providing a common language and platform for building collaboration internally and with external partners”.

Both the University of Sydney and Doctors for the Environment Australia point to the Planetary Health paradigm as best offering a framework for government to bring interdisciplinary approaches and partnerships to realization. Alternatively, the International Women’s Development Association commends “A focus on gender equality and women’s empowerment” would provide government with the framework “for moving away from a siloing of the goals towards an approach driven by values and crosscutting priorities”.

#### Data strengthening for robust data and strong evidence base for health

DFAT notes “Australia is working to continuously improve data collection” including “supporting developing country partners to strengthen their statistical capacity, and engaging in initiatives to improve data collection”. Certainly, many of the stakeholders emphasize that SDG success in Australia and the Indo-Pacific region will greatly depend on robust data generated by strong and reliable information systems, and the use of such data as evidence in SDG decision-making policy and planning. Consequently, many stakeholders advocate, “The collection of high-quality, timely and disaggregated data should be prioritized to enable the Australian Government to track the experiences of vulnerable groups such as children” [World Vision Australia].

UNICEF Australia points out that although governments who receive Australian aid are “ultimately accountable to generate the data that will guide and measure achievement of the SDGs”, the Australian Government “and the international community more broadly, has an obligation to partner with these countries to make sure the SDG targets are met”. The Australian Council for International Development recommends Australia “Invest in new measurement capacity to strengthen the collection of disaggregated data, and continue to support capacity building in this area in the Pacific”, including supporting and building “the capacity of National Statistics Offices in our region to collect disaggregated and intersectional data and to facilitate sharing of common learnings regionally to enable regional reporting on SDGs, reduce the burden on National Statistics Offices, and identify issues and trends relevant across countries”.

It follows that for a number of Inquiry stakeholders, strong health and wellbeing data is particularly critical in light of health inputs and outcomes cross-cutting all 17 SDGs, and specifically gender-related and SRH data: “it is crucial that Australia’s ODA program improves the poor SRH data collection across the Pacific so a fuller understanding of need can be gained” [Family Planning NSW]. Indeed, several respondents identified the lack of gender-disaggregated data is a major challenge in the Pacific region, which will impede countries ability to measure SDG progress, including health progress.

### Theme 3: Australia’s regional and global health priorities

Inquiry respondents emphasize the Australian Government should advance the health of priority populations in its ODA-SDG efforts in the Indo-Pacific region, and elsewhere. The health and wellbeing of five priority populations are identified: children; adolescents; persons with disabilities (explicitly including persons with vision impairment); ‘Left Behind’ marginalized populations Left Behind; and women and girls (Table 3).

**Table 3:** Population segments of focus in Australia’s ODA-SDG health and well-being investment efforts

Five priority populations	Rationale
1. Child health and wellbeing	• “The 2030 Agenda presents an opportunity to support the rights and best interests of all children. It is a chance to provide children with the best start in life, and to ensure that they survive and thrive. The 2030 Agenda is a comprehensive and holistic framework that creates a vision for the world where children can live free from violence and abuse and in an environment that supports their healthy development” – UNICEF Australia • “One practical way that the Australian Government can show leadership on leaving no one behind in the Indo-pacific region is by being an advocate for child protection and the elimination of violence against children” – World Vision Australia
2. Adolescent health	• “Current population specific funding mechanisms targeting key affected, vulnerable and marginalised populations still exclude adolescent populations as a demographic priority. Australian ODA should be consolidated to enable an inter-sectoral and multi-component engagement of key SDGs that will harness the opportunities of the triple dividend that benefits during adolescence, across the life course, and into the next generation” – Burnet Institute • “Adolescence is a pivotal period during which the gains of childhood can either be consolidated or lost. The second decade of life presents an opportunity to build on gains made in childhood and to invest in programmes that contribute to healthy, safe, informed and empowered transitions to adulthood. It can also be a forgotten stage” – UNICEF Australia
3. Persons with disabilities	• “Australia has a strong commitment and track record on disability inclusion in the aid program. This is demonstrated by DFAT’s international advocacy for disability inclusive development, the commitments set out in Australia’s *Development for All* strategy for strengthening disability inclusive development, and reported progress… Recommendation []: Inform all future program investments according to the Office of Development Effectiveness’ strategic evaluation of DFAT’s work promoting disability-inclusive development” - Oxfam Australia • “Importantly, [the SDG] document references disability 11 times… Given the Millennium Development Goals (MDGs) made no reference to persons with disabilities, this is an achievement in itself, presenting a tangible opportunity for the inclusion of people who are blind or vision impaired to be counted” – Vision 2020 Australia
4. Marginalized & poor populations	• “The two most frequently-used words in the description of the SDG targets are “for all”. To improve the lives of everyone, care must be taken to ensure those who are most disadvantaged benefit from SDG progress… Australia’s domestic and international policies therefore need to prioritise marginalised and vulnerable groups, including but not limited to women, children, people with a disability, Indigenous people and ethnic and sexual minorities… The principle of leaving no one behind equally applies to Australia’s work overseas” – World Vision Australia • “Ensure Australia’s approach to implementing the SDGs domestically and through our aid program centres on the commitment to leave no one behind, with concrete strategies to support inclusion of disadvantaged groups in the design, delivery and monitoring/evaluation and reporting on services and programs. This should result in…explicitly targeting the poorest communities and most marginalised groups within the countries where Australian aid is directed to... Although the drivers of inequality are complex and multi-dimensional, evidence shows that it is necessary to focus on the needs of the most disadvantaged populations to reduce inequality… Accordingly, it is important for Australia to make strategic choices about the type of aid it provides in support of poverty reduction and addressing inequality. Priority must be given to investing in human capital as a foundation for inclusive, equitable and sustainable development” – Save the Children
5. Women and girls’ health and wellbeing, and gender empowerment	• “It is critical for Australia to support integrated, multi sectoral approaches that empower women and girls and break the intergenerational cycle of poverty. We welcome the Australian Government’s strong commitment towards greater gender equality, as reflected in the goal that 80% of Australia’s development program will address gender issues in implementation” – Save the Children • “Recommendation []: Australia should retain the 80% gender target, and ensure this target is made more meaningful by: o Increasing transparency of DFAT’s self-assessment process so that stakeholders understand how programs are being assessed; o Ensuring all partners, including private sector partners, integrate gender analysis from design through to program evaluation; o Ensuring DFAT has sufficient technical expertise in gender mainstreaming to accurately assess all programs; o Increasing financial expenditure on programs were gender equality is the principal objective; and o Increasing transparency of the financial allocation towards gender equality in programs where gender is a significant objective” – Oxfam Australia

Further, six key health issues for SDG health and ODA prioritization by the Australian Government are identified in the submissions included in our analysis (Table 4). The first pertained to the Australian Government continuing to invest in health system strengthening efforts, especially in terms of supporting inter-related Universal Health Coverage (UHC) strengthening, such as research. The second health priority identified is SRH, followed by combatting communicable and infectious disease, addressing the social determinants of health, poor eye health, and health security.

**Table 4:** Six priority issue areas for ODA-SDG Investment in Health

Six priority issue areas	Rationale
1. Health system strengthening	• “Progress in generating research evidence to support UHC has been uneven, and investment in low-income countries’ research production neglected. Currently, only 10% of health policy and systems research globally is conducted on low- and middle-income countries. Building the capacity of poorer communities to research and learn is key to their sustainable development” – Burnet Institute • “To implement sustainable change in the Pacific health sector, long term investment is required to build the capacity of Pacific communities to implement change themselves. Investment in infrastructure to provide appropriate health facilities is essential. Critical to achieving improved health outcomes is training of clinicians, community workers and teachers, trialling and implementing customised programs, and ensuring that they continue by embedding teaching in schools and universities” – Family Planning NSW • “[Recommendation] Scale up ODA investments in strengthening health systems to support the provision and expansion of Universal health Coverage (UHC) schemes, including the provision of high quality, comprehensive and integrated eye care services” – Fred Hollows Foundation • “Using an inclusive approach to intervene at multiple levels of the health system with locally tailored responses to eye health workforce development increases the efficiency, suitability and sustainability of the investment by the Australian Government, provides the best chance to leave no one behind” - Vision 2020 Australia
2. Sexual and reproductive health (SRH)	• “The unmet need for family planning remains unacceptably high, especially in disadvantaged populations and in under-developed and developing countries, including in the Pacific region. In the Solomon Islands, for example, population increases are expected to outstrip food and water supply within ten years. Neighbouring Papua New Guinea is experiencing similar critical sustainability issues in relation to its current population projections. These parts of the Indo-Pacific are faced with limited and unreliable contraceptive supply, with some of the most under-resourced reproductive services in the world” - Family Planning NSW • “We further recommend that our aid program continue to support the provision of SRH services in humanitarian emergencies and prioritise SRH support for regions where maternal mortality and the need for contraception is highest. Access to quality reproductive services has a transformative impact on women’s health, education and empowerment and is therefore essential to gender equality” - Australian Parliamentary Group on Population and Development • “[Recommendation] The Australian Government [] increase its current funding of $23.7 million to investing $50 million per annum for reproductive health initiatives” – Global Citizen Australia • “Sexual and reproductive health is an area of considerable success and great potential. DFAT’s *Gender Equality and Empowering Women and Girls* strategy recognises reproductive health as a key factor in gender equality and economic empowerment, and the *Family Planning and Aid Program – Guiding Principles* are supportive of comprehensive care. Family planning is a mechanisms for achieving the SDGS as it contributes to decreasing maternal and child mortality and poverty, achieving gender equality, sustainable cities, responsible consumption, decent work, and access to higher education. Family planning can also help mitigate climate change and promote sustainable communities” – Marie Stopes International Australia
3. Combatting communicable and infectious disease	• “As part of an increase in development assistance for health, Australia should focus on… Increased assistance to combat the infectious diseases claiming the most lives – HIV, TB and malaria – both through bilateral assistance in our region and through the Global Fund to Fight AIDS, TB and Malaria; Increased action to ensure all children have access to vaccines, including through ensuring the re-use of resources from the polio eradication campaign to improve vaccination systems” – Results International Australia
4. Social determinants of health, such as nutrition and food security, education, climate change & environmental health	• “Undernutrition is widespread in countries in Asia and the Pacific, in spite of their economic progress. Taking further action on nutrition would be consistent with Australia’s aid objectives of promoting sustained economic growth, improving health and education, and empowering women and girls – Results International Australia • “The impact of under nutrition on the health, productivity and survival of individuals should be of concern for Australia given that its neighbours in the Pacific have some of the highest child undernutrition rates in the world. In particular, in Papua New Guinea (PNG) almost one in two children are stunted from undernutrition – the fourth highest child stunting rate in the world. In a ground-breaking report released in 2017, Save the Children and Frontier Economics estimated that child undernutrition cost the PNG economy a staggering $1.5 billion (8.45% of GDP) in a single year. Yet only 0.1% of Australia’s [ODA] to PNG was allocated to nutrition in the years 2010 and 2012 (latest data publicly available). It is not possible to promote inclusive and sustainable economic development in the long term in PNG if around half of the population of working age continues to suffer reduced productivity from childhood undernutrition. Indeed, child undernutrition will likely impede the potential impact of other aid investments hat bilateral and multilateral donors make for the purpose of promoting economic growth” – Save the Children • “[Recommendation] The Australian Government should increase its support for education from 18 to 20% of the Australian aid program and increase its share of total funding for the Global Partnership for Education… In relation to Goal 3, Ensure healthy lives and promote well-being for all at all ages, achieving universal education could: Prevent seven million cases of HIV and AIDS in the next decade; enable women with at least 7 years of formal education to have two or three fewer children; and reduce child mortality rates” – Results International Australia • [Recommendation] The Australian Government to increase its support for education and increase its share of total funding for the Global Partnership for Education from current investment of $900 million to $200 million AUD” - Global Citizen Australia • “[Recommendations] Australia should increase its contribution to international climate finance as part of a growing aid program and in line with Australia’ international obligations; Australia should develop a comprehensive Climate Change Strategy for the aid program” – Oxfam Australia • “As our closest neighbours in the Pacific are among those most vulnerable to climate change and associated health impacts, Australia should be positioning itself to work on all SDGs in ODA but with a focus in the region on climate change, clean energy, land and ocean use, and chronic diseases and gender equality” – Doctors for the Environment Australia
5. Eye health	• “[Recommends] “Eye health and vision care is regarded as a public health priority in Asia and the Pacific” – Vision 2020 Australia • “Good vision transforms lives and has positive development impacts far beyond good health; it can enable individuals and families to pull themselves out of poverty, helps people to go back to work or school, and to overcome inequality, marginalisation and exclusion that blindness and vision loss often perpetuate” – Fred Hollows Foundation
6. Health security	• “DFAT engagement that aligns with SDGs 3, 5, 16 and 17[:] Australia’s *Foreign Policy White Paper* highlights our commitment to guarding against global health risks, in particular preventing and responding to the introduction and spread of infectious diseases. The Minister for Foreign Affairs recently launched the Indo-Pacific Health Security Initiative ($300 million, 2017–22), which will support efforts to prevent and contain disease outbreaks in the Indo-Pacific that have the potential to cause social and economic impacts on a national, regional or global scale. Complementary to this initiative, Australia’s ODA program supports countries to build strong, functioning health systems, which are critical to promoting sustainability and achieving sustainable economic growth” – Department of Foreign Affairs and Trade (DFAT) • “The new Indo-Pacific Centre for Health Security is a very welcome initiative and a clear opportunity for Australian ODA to support progress in areas of health and wellbeing (SDG 3), both short-term through infectious disease and biosecurity and in the long-term by addressing drivers of risky behaviour at the individual, household, community, national and regional levels” – University of Sydney

### Theme 4: financing to deliver regional and global health SDG imperatives (and the SDGs generally)

Stakeholders had much to say about ODA-SDG financing, governance and policy. Repeated calls are made for the Australian Government to: increase its investment in ODA (including health-related ODA); alter Australia’s ODA policy to overtly align with the SDGs; improve government transparency around ODA expenditure; support stronger tax collection processes for unlocking finance for SDG achievement in countries recipient of Australian aid; and leverage the Australian private sector to partner and invest in ODA-SDG opportunities, such as impact investment.

#### Strong calls for Australia’s investment in ODA, including ODA for health programs, to be increased

Although stakeholders acknowledge that ODA is one part of the solution to raising development finance for the SDGs, it will nonetheless “play a vital role” [World Vision Australia]. Most stakeholders are critical of the current level of Australian ODA and call on the Federal Government to rebuild the country’s ODA budget for the Indo-Pacific region. There were differing suggestions on aid targets. According to the Burnet Institute, for example, “The Australian Government should reinstate – and seek bipartisan support for – the goal of the Australian aid budget reaching 0.5% of GNI by a set date, at least before 2025” . World Vision Australia recommends rebuilding the aid budget “back to 0.33% of Gross National Income (GNI) over six years (2023-2024), and continue to incrementally increase funding to deliver on Australia’s international commitment as embodied in the SDGs, noting the 2030 Agenda encourage[s] developed countries like Australia to invest 0.7% of GNI in aid”. Results International Australia suggests ODA be increased “to 0.3% [GNI] over the next four years, and to 0.48% of GNI (returning to the historic peak of Australian aid) within 10 years”.

A number of Inquiry respondents express concern that if Australia’s ODA expenditure is not increased, Australia’s impact and influence in regional SDG achievement – and leadership in the Indo-Pacific more broadly - will be compromised:

“[T]he diminished funds available for aid have severely constrained DFAT’s ability to effectively respond to the challenges of achieving the SDGs. Newer initiatives are fewer in number, with little opportunity to build upon notable successes in health and education from the previous decade. Since FY2013-14, the aid budget has been cut by nearly 25% in total and the forward estimates that once outlined the growth trajectory to reaching 0.55% GNI in 2020 have been repeatedly reversed.” [Burnet Institute].

For Doctors for the Environment Australia, ODA cuts are “detrimental to regional solidarity and sustainable development”, and as the nation “drops out of the OECD club of top ten donors in rankings of global donor generosity – it will lose influence on issues and values that Australia traditionally leads or holds expertise in”. Save the Children also emphasize geo-political reasons for the Australian Government to increase its ODA investment: with the rise of emerging economies in the Asian region that are increasing their aid spending (, Australia faced increased competition for access and influence in the global economy as well as global decision-making forums. Therefore, increased investment in ODA could extend Australia’s geo-political influence.

“We appreciate that tough fiscal trade-offs need to be made to strengthen the Australian economy and return the budget to surplus. However, the drastic cuts made to Australia’s overseas aid budget are short-sighted saving measures… These [ODA] cuts have come at a cost to our international reputation, geo-political influence and capacity to credibly shape development outcomes in our region and beyond. Most crucially, these cuts have impacted the world’s poorest people, leaving the most vulnerable further behind.” [Save the Children].

In addition to the many broad calls for the Australian Government to increase its ODA-SDG expenditure, specific request is made for there to be an increase in Australia’s ODA health budget. The Burnet Institute considers that Australia’s’ ODA health budget should at least increase to pre-2014/2015 ODA levels:

“The aid budget increased slightly in FY2017-18 and FY2018-19 to $3.9 billion and $4.0 billion, respectively; however, the steep decline in the previous four years has disproportionately affected health and education. For example, between the 2014/15 and 2015/16 budgets…The health budget fell by $221 million or 28%. Between them, education and health made up 37% of the aid program before 2015/16, but contributed 52% of the cuts from that year. Both sectors are crucial for long-term development, economic growth and regional security.”

Vision 2020 Australia points out that “public health interventions require long-term commitment which goes beyond short term policy and political cycles” and therefore recommends the Australian Government “Commit to long term funding for [the] SDGs”. Meanwhile, the Fred Hollows Foundation advocates the Federal Government “Embed the principles and commitments of the Addis Ababa Agenda for Action in Australia’s ODA program and accelerate efforts to realize Australia’s commitment to increase the aid budget to 0.7% of [GNI] by 2030 through a sustained and predictable trajectory”.

Improvement in Australia’s ODA investment and resourcing in SRH is additionally called for in light of Australia halving its investment in family planning within the aid program from $46.4 million in 2013/14 to $23.7 million in 2015/16 [Family Planning NSW; Marie Stopes International Australia]. Increased investment in nutrition programs in the Pacific is further advocated, “Australia should commit at least 3% of ODA on nutrition-specific interventions (as defined by OECD-DAC criteria) and nutrition-sensitive interventions… including targeted support for the Pacific” [Results International Australia], as well as investment in the environmental determinants of health, including climate change [Oxfam Australia].

#### Changing the rhetoric and focus of Australia’s ODA program to align with the SDGs

Not only do Inquiry respondents promote increased investment in various ODA health programs, but several stakeholders contend the entire focus of the Australian Government’s ODA program should change from a “trade-for aid approach to development” to one that reflects the “broader aims articulated under the SDG framework for achieving development” [Victoria University]. For Save the Children, this would involve improving the targeting of aid to population segments within the Indo-Pacific region in line with the SDG’s Leave No One Behind principle, including “Improving the targeting of development assistance sub-nationally to reach geographic areas and disadvantaged population groups”. The International Sexual and Reproductive Health and Rights Consortium also calls for the targeting of Australian aid investment in SRH services to the countries and populations where needs are greatest. These include “the 12 countries in our region that are home to 90 per cent of maternal deaths”, and “rural communities, ethnic minorities, people living with disability, sex workers, and people who may face persecution or stigmatization due to their gender identity or sexual orientation”.

#### Australian government investment in improving its ODA reporting and the enabling of countries, which are recipients of Australian aid, to improve tax collection for SDG financing

The need for greater transparency and accountability in the Australian Government’s acquittal of ODA expenditure is emphasized by several stakeholders. For this to occur, Oxfam Australia and World Vision Australia suggest the government increase its annual investment in resourcing the Office of Development Effectiveness, “to expand its mandate to regularly assess the Australian aid program against the SDGs and identify and disseminate best practice examples”. Save the Children held parallel concern around a decline in Australian aid transparency and made several recommendations, which include follow the UK’s Department for Foreign International Development’s lead in reporting on aid allocations using the International Aid Transparency Initiative) open standard, and for the Australian Government to reinstate the aid ‘blue book’ to be published alongside other budget documents.

Allocating additional ODA to address both multinational tax avoidance and build the tax collection capacity of countries that received Australian aid to improve good governance and unlock further financing for the SDGs is advanced by Oxfam Australia. The latter recommends the government: “Focus more foreign aid efforts on building the tax collection capacity of recipient countries’ tax administrators – in line with Australia’s commitment under the Addis tax initiative –to doubling our support in the area of taxation and domestic resource mobilization in developing countries from $16 million in 2014-15 to $32 million by 2020”.

#### Leverage Australian private sector investment in ODA-SDG strategy and activities

Many of the stakeholders in their submissions to the Inquiry acknowledge that government funding “would not be sufficient to meet the ambitious aim of leaving no man, woman or child behind” [Save the Children]. For Cardno Development International, the private sector must therefore be more effectively engaged in ODA. Accordingly, “the Australian Government [needs] to incentivize and accelerate the establishment of new models to leverage private capital flows together with ODA to enable both commercial and development dividends” [Save the Children]. The Australian Council for International Development agrees, recommending the government “Adopt new and innovative blended finance approaches that allow Australia to leverage investment from multiple sources to achieve the SDGs”*.*

Regarding such new models, World Vision Australia welcomes the Australian Government’s 2017 announcement to establish a $40 million Emerging Markets Impact Investment Fund (EMIIF) to support investment in small and medium-sized enterprises in the Asia Pacific region. In turn, World Vision Australia fears “it is not yet clear which SDGs the EMIIF will contribute to other than those directly related to economic growth through a gender lens”. It thus proposes that the EMIIF is complemented by a $100 million Sustainable Development Impact Fund “to augment its aid and social services funding by providing finance to the private sector for investments that promote sustainable development in Australia and in aid recipient countries”. World Vision Australia also recommends that a Sustainable Development Impact Fund use “an impact investing model aimed at incentivizing private sector investment to advance the SDGs in Australia and overseas”.

World Vision Australia further praises the business-led Global Compact Network Australia (GCNA) to advance the private sector’s contribution to the SDGs, including its launch of the CEO Statement of Support for the SDGs in 2016, and its recent launch of an online Australian SDGs Hub for Business. Doctors for the Environment Australia further recommendthat the GCNA and its business alliances “be directly informed by health and environment communities” to “allow tracking of progress as well as harms to health”:

“The [GCNA] is increasingly recognizing the historical harms to public health by certain actions of the corporate community (i.e. tobacco and anti-retroviral pricing). National businesses declaring commitment to the SDGs can better align with the health and environment community on health and environment themes to forge and expedite different healthy futures”.

In terms of advancing public private partnerships for SDG achievement in health, the Burnet Institute suggests the Australian Government take part in the Bill and Melinda Gates Foundation’s expanded Grand Challenges in Global Health initiative, as had Canada, South Africa, Brazil, India, China and Korea. For the Burnet Institute, Australia could especially progress a Grand Challenges program similar to Grand Challenges Canada, a multi-stakeholder fund led by the Government of Canada: “Since 2015, Grant Challenges Canada has explicitly aligned its objectives with the SDGs, which focuses the use of research and innovation on those global targets”.

### Part 2 - comparison of whether, or to what degree, the above four themes are reflected in the FATRC summary of the submissions of early 2019 and Australia’s SDG VNR (June 2018)

#### FATRC report comparison

We found limited congruency exists between the findings from the first part of our study and the content of the FATRC summary of the content of the Inquiry submissions released in February 2019. The content of only one of our four thematic findings, Theme 2, is substantively reflected in the report’s 18 Recommendations. Our Theme 2 (importance of governance, policy coherence and planning for integrating SDG implementation into Australia’s ODA program) does resonate with four of the FATRC’s 18 Recommendations:

- Recommendation 1 - The Australian Government develop a national SDG implementation plan;
- Recommendation 4 - The SDGs are integrated in all Australian Government agencies’ policies and strategies;
- Recommendation 9 - Government should improve SDG awareness among all stakeholders; and
- Recommendation 18 - Continue SDG integration into Australia’s ODA program and prioritize the commitment to leave no one behind.

Although the FATRC report recommends the Australian Government “assesses opportunities to encourage sustainable public procurement, impact investment and business practices that support the [SDGs]” (Recommendation 17), this recommendation nonetheless falls short of the recommendations given by Inquiry stakeholders in our Theme 4 on financing for the goals. Inquiry stakeholders do not suggest the Australian Government should “assess[] opportunities” with the private sector for ODA-SDG implementation. Rather, stakeholders in our analysis strongly recommend the Australian Government actively pursue and leverage the private sector for ODA-SDG financing opportunities in addition to the Australian Government increasing its expenditure on ODA, including health-related ODA.

Nor did the FATRC final report of February 2019 deal with SDG 3 priorities, or other inter-related SDG health and wellbeing priorities, in any depth - including in report recommendations. This is despite the Inquiry’s Terms of Reference specifically asking respondents to comment on what SDGs are currently being addressed by Australia’s ODA program (Terms of Reference question e); which of the SDGs is Australia best suited to achieving through our ODA program, and should Australia’s ODA be consolidated to focus on achieving core SDGs (Terms of Reference question f).

#### SDG-VNR comparison

The Australian Government’s treatment of ODA and health and development in its SDG-VNR of June 2018 comprises one-and-a-half pages of its four-page narrative on SDG 3. Consistent with the 2017 Foreign Policy White Paper, the government reports it is utilizing the SDG agenda to advance Australia’s ongoing national needs, notably progression of the government’s health security agenda. Australia’s SDG VNR specifies this will include Australia guarding against regional and global health risks (notably mosquito-borne viruses such as malaria, zika, dengue and chikungunya, and infectious disease), investing in research partnerships through the Indo-Pacific Centre for Health Security, as well as preventing, detecting and responding to health emergencies and tackling antimicrobial resistance. Australia’s commitment to regional health systems strengthening is also acknowledged in the VNR, as is brief acknowledgement of the impact of climate change on health and wellbeing in the Pacific: “Our neighbours in Pacific small island states face specific health care challenges, including high rates of non-communicable diseases (NCDs), geographic barriers, constrained health spending and health sector workforce shortages and health risks posed by climate change” [10]..

We further found the VNR addresses some of the issues stakeholders identify that are outlined in Part 1 of the research findings. The VNR’s Global and Regional Action priority areas for health overlap with two of the six health priority issues identified in Theme 3 of our analysis - (1) combatting communicable and infectious disease and (2) health security. Although the VNR mentions strengthening primary health care in its section on Health Challenges in the Pacific, there is not an emphatic embrace of the pursuit of health system strengthening in the region as pressed by this study’s stakeholders (see Table 4). The VNR also highlights the high rates of NCDs in the Pacific context, which aligns with our study’s stakeholders prioritization of the social determinants of health to combat chronic disease (Table 4).

Strong written advocacy by vision impairment agencies in the Parliamentary Inquiry ensure the inclusion of eye health in our analysis as a key ODA-SDG priority area for health action in the Indo-Pacific. However, eye health is overlooked in Australia’s VNR. Stakeholder SRH advocates also identify action on SDG 5 (Achieve gender equality and empower all women and girls) as a priority area for the Australian Government’s ODA-SDG activities in our analysis. However, in the VNR, the Australian Government’s role in advancing SRH in the Indo-Pacific region is not overtly but implicitly mentioned by reference to the funding of SRH agencies: “We are a long-standing, major donor to global funds and organizations, including the Global Fund to fight AIDS, Tuberculosis and Malaria, Gavi the Vaccine Alliance, UNAIDS and the United Nations Population Fund (UNFPA), as well as multilateral banks that work in the health sector”.

## Discussion

This study does not offer an analysis of the Australian Government’s ODA policy as it engages the SDGs in the Indo-Pacific region. What it does do is explore the dialogue around those issues, at the invitation of the Parliamentary Inquiry into the UN SDGs, examining and synthesizing the responses of selected Inquiry stakeholders. The study then compares the stakeholder priorities and concerns on ODA-SDGs, health and development with the content of the government’s SDG-VNR, as well as with the FATRC report on UN SDG Inquiry findings. This study finds there is limited resonance in both the VNR document, and the Senate’s reporting of the Inquiry findings, with the views offered by multi-sectoral stakeholders whose written submissions to the Inquiry engage with the ODA-SDG and health and development intersection.

It is also unhelpful that the Australian Government released its first SDG-VNR in mid-2018 prior to the completion and reporting of the country’s Parliamentary Inquiry into the UN SDGs, even if the content of the FATRC’s final report into that Inquiry is less than comprehensive in its discussion of all eight of the Inquiry’s Terms of Reference as per this study’s findings. While we acknowledge that the Australian Government notes in its SDG-VNR that it did indeed conduct VNR consultations throughout 2017–2018, and a lengthy list of consulted organizations and contributors are thus listed in the SDG-VNR, we nonetheless contend that by releasing its VNR before critically sifting through the 164 written submissions to the Inquiry offered by engaged multi-stakeholders, the government put the proverbial cart before the horse. In this regard, the Australian Government certainly missed an opportunity to systematically and transparently engage with an important and useful evidence base for SDG policy making and planning – the voices of Australian government and non-government agencies, industry and business, civil society, and the Australian people – which would have made engaging in the Inquiry process more cost-effective and impactful. It would seem this is an example of ‘policy based evidence making’ instead of ‘evidence based policy making’ that Pankhurst suggests arises in cases involving governmental policy resolution of wicked problem s[39]..

It follows that the four thematic findings derived from the submission data present two key messages to the Australian Government. The first message draws attention to the critical role of health in moving Agenda 2030 forward in the Indo-Pacific region, as Australia’s ODA-SDG program engages regional and global health priorities, including the health and wellbeing of five priority populations identified by the selected Inquiry stakeholders (Themes 1 and 3). The second message is broader and structural in nature, and pertains to the importance of good governance, policy coherence and planning mechanisms for Australia’s ODA-SDG roll-out, which includes improving financing and aid accountability and transparency practices for implementing the SDGs (including SDG 3) (Themes 2 and 4). A comparison with Australia’s VNR released in June 2018, and the FATRC’s summary of the Parliamentary Inquiry submissions completed in 2019, suggests that these messages remain pertinent.

The Australian Government’s recent renewed interest in both ODA and the SDGs has not gone unnoticed either domestically or internationally, or in the Pacific region especially. The reason for this renewal is manifestly clear: Australia’s security agenda vis-à-vis Chin a[20–22]. Increasingly, Australia’s regional policy towards its Pacific neighbours is being driven by concerns of growing Chinese presence in the Pacific region, and the desire for influence in the islands to the North and Northeast of Australia; an imperative which has always been central to Australian geopolitical though t[17, 18]. Thus, on the one hand, renewed Federal Government interest and stakeholder engagement is a positive step and the evidence from this paper shows that Australian SDG stakeholders are deeply committed to improving Australian ODA, especially in the context of the fulfilment of Australia’s ODA-SDG commitments. However, the government’s own policies and behaviour may be working against this goa l[32]. As the results of this study found, there is a lack of correspondence between stakeholder engagement and government thought as demonstrated by the VNR and FATRC reports. If Australia wishes to improve the effectiveness of its ODA then it follows that listening to those Pacific nations, their leaders and their diverse peoples, as well as those heavily involved in the health and development space, is beneficial.

## Conclusion

The voices, knowledge and guidance provided by the multi-stakeholders included in this research reaffirm that the SDG agenda presents an enormous opportunity to be both the catalyst and the vehicle for enabling a paradigm shift in the Australian Government’s development approach toward the Pacific and its diverse peoples. A reframed health and development policy commitment that is embedded in an inclusively devised Australian ODA-SDG program could be a key part of that catalytic shift. However, the SDGs cannot be achieved in Australia and Indo-Pacific region by government action alone - despite the primacy of the Australian Government’s UN SDG commitment. The stakeholder submissions suggest that there is an interest across the sectors to be engaged both in discourse and action — for the SDGs to be achieved, that interest needs to be leveraged and extended, and ongoing, open debate and overt planning, is crucial.

Of concern, guidance around critical health and development ODA-SDG issues from the rich and diverse Inquiry stakeholder voices that came to life through this research are neither adequately captured in Australia’s SDG-VNR or Senate report that distilled submission input. This emphasizes that documentary analysis of public records remains a useful tool for health policy researchers examining policymaking processes and their surrounding complexities,[40] and that such researchers have a role to play in accountable and transparent evidence-based policy translation by government.

Although the findings of the first part of this study provide useful suggestion to the Australian Government on what to do and how to do it in the context of Australia’s ODA-SDG health and development rollout, the findings from the second part of this study remind that at the heart of Australia’s ODA-SDG health and development commitment and practice are found the political determinants of health and the political determinants of the SDG agend a[41–43]. Having cogent understanding of both sets of determinants, and their overlap, cannot be under-estimated.
